# Calcium/calmodulin-dependent protein kinase II associates with the K^+^ channel isoform Kv4.3 in adult rat optic nerve

**DOI:** 10.3389/fnana.2022.958986

**Published:** 2022-09-08

**Authors:** Genki Ogata, Gloria J. Partida, Anna Fasoli, Andrew T. Ishida

**Affiliations:** ^1^Department of Neurobiology, Physiology, and Behavior, University of California, Davis, Davis, CA, United States; ^2^Department of Ophthalmology and Vision Science, University of California, Sacramento, Sacramento, CA, United States

**Keywords:** Kv4.3, CaMKII, 4-aminopyridine, co-immunoprecipitation, immunohistochemistry, conduction velocity, axon, retinal ganglion cell

## Abstract

Spikes are said to exhibit “memory” in that they can be altered by spikes that precede them. In retinal ganglion cell axons, for example, rapid spiking can slow the propagation of subsequent spikes. This increases inter-spike interval and, thus, low-pass filters instantaneous spike frequency. Similarly, a K^+^ ion channel blocker (4-aminopyridine, 4AP) increases the time-to-peak of compound action potentials recorded from optic nerve, and we recently found that reducing autophosphorylation of calcium/calmodulin-dependent protein kinase II (CaMKII) does too. These results would be expected if CaMKII modulates spike propagation by regulating 4AP-sensitive K^+^ channels. As steps toward identifying a possible substrate, we test whether (*i*) 4AP alters optic nerve spike shape in ways consistent with reducing K^+^ current, (*ii*) 4AP alters spike propagation consistent with effects of reducing CaMKII activation, (*iii*) antibodies directed against 4AP-sensitive and CaMKII-regulated K^+^ channels bind to optic nerve axons, and (*iv*) optic nerve CaMKII co-immunoprecipitates with 4AP-sensitive K^+^ channels. We find that, in adult rat optic nerve, (*i*) 4AP selectively slows spike repolarization, (*ii*) 4AP slows spike propagation, (*iii*) immunogen-blockable staining is achieved with anti-Kv4.3 antibodies but not with antibodies directed against Kv1.4 or Kv4.2, and (*iv*) CaMKII associates with Kv4.3. Kv4.3 may thus be a substrate that underlies activity-dependent spike regulation in adult visual system pathways.

## Introduction

Calcium/calmodulin-dependent protein kinase II (CaMKII) is present in the dendrites, somata, axons, and axon terminals of many neurons. Presynaptic and postsynaptic roles of CaMKII in synaptic plasticity have been widely studied (Lisman et al., 2002), and CaMKII has been found to regulate signal spread in dendrites (Tsubokawa et al., 2000). By contrast, little is known about CaMKII effects on signal propagation in axons, although CaMKII has been detected in the axons of various central neurons (Ouimet et al., 1984; Terashima et al., 1994; Lund and McQuarrie, 2001). We have previously shown that CaMKII autophosphorylated at threonine 286 (pT286) is present in optic nerve axons, and that electrical stimulation reduces optic nerve pT286 levels and increases the time-to-peak of optic nerve compound action potentials (Partida et al., 2018). As might be anticipated, we also found that the time-to-peak of optic nerve compound action potentials is increased by an inhibitor of Ca^2+^/CaM binding to CaMKII (KN-93) and decreased by phosphatase inhibitors (okadaic acid and fostriecin; Partida et al., 2018). These results are consistent with the possibilities that spiking slows the propagation of subsequently elicited spikes (Raymond et al., 1990; Meeks and Mennerick, 2007) and that this effect arises, at least in part, from reducing the level of activated CaMKII (Yamagata and Obata, 1998; Menegon et al., 2002).

Previous studies have not identified what CaMKII regulates in axons in order to alter spike propagation. At least one substrate might be K^+^ channels, based on the finding that 4-aminopyridine (4AP), like KN-93, increases the time-to-peak of optic nerve compound action potentials [Foster et al., 1982; see also the recordings in Brown et al. (2001) and Devaux et al. (2002)]. It might seem unlikely that 4AP and KN-93 produce similar effects, because 4AP is commonly used to inhibit current flow through depolarization-activated K^+^ channels (Coetzee et al., 1999) and this differs from the effect of KN-93 on CaMKII (Sumi et al., 1991). However, studies in heterologous expression systems and smooth muscle have shown that K^+^ channels are modulated by KN-93 and by CaMKII (Roeper et al., 1997; Koh et al., 1999), and studies of *Drosophila* heads and cardiac myocytes have found that some molecular subtypes of K^+^ channel complex or associate with CaMKII (Sun et al., 2004; Colinas et al., 2006). Whether similar modulations and associations occur in optic nerve has not been tested to date, and a molecular subtype of K^+^ channel that associates with optic nerve CaMKII has not been identified. The present study begins to address these questions in four steps.

First, we ask if 4AP slows spike repolarization, as would be expected if 4AP reduces outward K^+^ current as in other tissues (Storm, 1987). Second, we ask if 4AP slows spike propagation, as proposed for KN-93 (Partida et al., 2018). Third, we ask if antibodies directed against 4AP-sensitive and CaMKII-regulated K^+^ channels bind to optic nerve axons. Fourth, we ask if CaMKII co-immunoprecipitates with the subtype of K^+^ channel we immunolocalize in optic nerve axons. Answering the third and fourth questions depends on answers we obtain to the first and second questions. However, answering the first two questions by use of compound action potentials is hindered by two properties of their waveforms. One is that optic nerve compound action potentials display multiple peaks (cf., Bishop et al., 1953). The second is that these peaks partially overlap (Stys et al., 1991). Because each peak is formed by spikes propagating in multiple axons at similar but not identical speeds, the rate at which each peak rises and falls is weighted by increases and decreases in the number of spikes propagating at different speeds. Because the cardinal peak starts from baseline, and the next peak starts to rise before the cardinal peak returns to baseline, the most rapidly propagating spikes can be timed by the base of the cardinal peak, but the start of each post-cardinal peak is hidden. We therefore record spikes in the present study with extracellular electrodes that can resolve their rate of depolarization separately from their repolarization, and that can more clearly show the time that elapses as spikes propagate between stimulating and recording electrodes. The results of these tests are of interest because activity-induced slowing of spike propagation can reduce the maximum spike frequency generated by axons (Partida et al., 2018) and this would modulate synaptic transmission to neurons in sub-cortical brain regions (Singer et al., 1972; Usrey et al., 1998).

## Materials and methods

### Animals

Adult Long-Evans rats (female; P60-P120; 150-250g) were obtained from a commercial supplier (Envigo Bioproducts) and housed in standard cages at ∼23°C on a 12-h/12-h light/dark cycle. For the experiments reported here, each rat was anesthetized by intraperitoneal ketamine and xylazine (70–100 mg/kg and 5–10 mg/kg, respectively; see below for the source of chemicals used in this study) and decapitated. The optic nerves were dissected and electrophysiologically recorded from, immunohistochemically stained, and western blotted, as described below. The results presented here are based on measurements obtained from a total of 35 rats (3 for spike recordings, 2 for immunohistochemistry, and 30 for western blots). All animal care and experimental protocols were approved by the Animal Use and Care Administrative Advisory Committee of the University of California, Davis.

### Spike recordings and analysis

**FIGURE 1:**
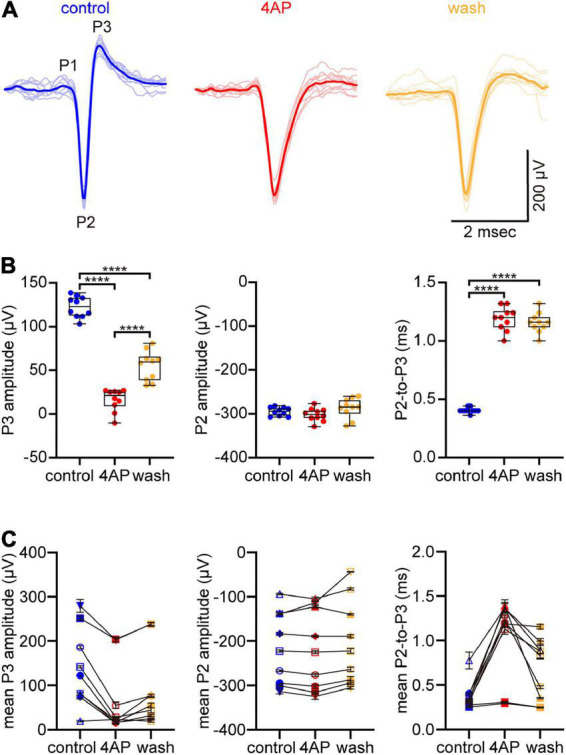
4-aminopyridine (4AP) slows spike repolarization without altering spike rate of rise. **(A)** Spikes that fired spontaneously at a multi-electrode array (MEA) electrode in control solution (left), 5 min after supplementing the superfusate with 1 mM 4AP (center), and 25 min after the superfusate was changed back to control solution (right). Ten spikes that fired in each solution are plotted in faint lines and superimposed over each other. An exemplar in each solution is highlighted by the strongly colored lines. The spikes are identified as axonal based on their triphasic waveform (initial upward peak denoted P1, downward peak denoted P2, subsequent upward peak denoted P3) in control solution. **(B)** Box plots of amplitudes of P3 (left) and P2 (center) of spikes in **(A)**, and of difference in time between these peaks (right) in control, 4AP, and wash solutions. Boxes and bars show median (intrabox horizontal line), interquartile range (IQR, box), and largest and smallest values no further than 1.5xIQR from each box (whiskers). Brackets above boxes point where the differences are statistically significant, with *n* = 4 asterisks signifying that *p* < 0.0001. **(C)** The type of amplitudes and differences plotted in **(B)**, recorded at a total of 9 electrodes in a total of 3 preparations. Each symbol plots the mean of *n* = 10 measurements. The error bars plot ± S.E.M., although some S.E.M. are smaller than the symbol. The values recorded at a given electrode in control, 4AP, and wash are connected by lines. Symbol shapes are arbitrary. The dots and symbols in **(B,C)** are color-coded like the traces in **(A)**.

**FIGURE 2:**
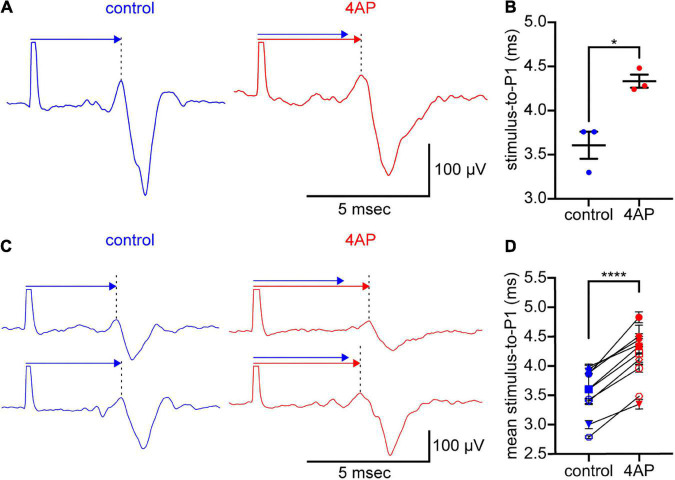
4AP slows spike propagation. **(A)** Spikes elicited by stimulation of an optic nerve and recorded intraretinally at an MEA electrode. The optic nerve was held in a suction electrode. The retina, attached to the optic nerve, was laid retinal ganglion cell-side down on the MEA. The spikes (traces below the arrows) were recorded in control solution (left) and 2.5 min after the superfusate was changed to solution containing 300 μM 4AP (right). Each trace is the digital average of 3 spikes recorded in each solution. The large upward deflection at the beginning of each trace is the stimulus artifact. These are cropped where they extend beyond the arrows. The spike waveform arrives later at the recording electrode. Above each trace, a like-colored arrow extends from the stimulus artifact to P1 (at the dotted vertical lines). A copy of the control arrow is plotted above the 4AP arrow to show the difference in spike propagation times. **(B)** Times elapsed in **(A)**, between the stimulus artifact and the P1 of 3 spikes recorded in each solution. Dots plot the times; wide horizontal bars plot the mean; error bars plot ± S.E.M. Difference between control and 4AP values is statistically significant (*p < 0.05). **(C)** Two rows of spikes, formatted as in **(A)**. Each row recorded in a different preparation than in **(A)**, before and during inclusion of 300 μM 4AP in the superfusate. **(D)** Times elapsed between the stimulus artifact and P1 of spikes recorded at a total of 10 electrodes in a total of 3 preparations. Each dot plots the mean of *n* = 3 measurements. Error bars plot ± S.E.M., although some S.E.M. are smaller than the dots. One pair of dots per electrode recorded from, connected by lines to show difference in values recorded at each electrode in control and 4AP. Differences between control and 4AP values are statistically significant (****p < 0.0001). Color-coding of voltage traces, arrows, labels in **(A,C)**, and values plotted in **(B,D)**, as in Figure 1.

For electrophysiological recordings, we isolated the retina from the eye without removing the optic nerve from the retina, laid the retina ganglion cell-side down on a multi-electrode array (MEA; Meister et al., 1994), and held the cut end of the optic nerve in a glass suction electrode. This allowed us to stimulate the optic nerve, record action potentials intraretinally (Marchiafava, 1976), and recognize the triphasic waveform of axonal spikes (Figures 1, 2). The initial, upward peak (hereafter denoted “P1”) of these spikes signals capacitive currents ahead of propagating spikes that depolarize axonal membrane (Meister et al., 1994). We use this as a time-point to compare the time required for spikes to propagate from the stimulating electrode to the recording electrode in different solutions (Figure 2). The second peak (hereafter denoted “P2”) is downward and prominent. It aligns temporally with the depolarizing phase of intracellularly recorded spikes, and its shape resembles the first time derivative of the depolarizing phase of intracellularly recorded spikes (Henze et al., 2000). The third peak (hereafter denoted “P3”) is upward. Its amplitude reflects spike repolarization, but its amplitude and shape can also vary from cell-to-cell with recording location, cell morphology, and ion channel distribution (Henze et al., 2000). We therefore use changes in the amplitude of P3 to gauge changes in repolarization on a cell-by-cell basis.

Before commencing data collection, the preparations were equilibrated to Ames medium by continuous superfusion for 1 h. Spikes were then elicited until their amplitude and waveform reached stable values while superfusing with control Ames medium, Ames medium supplemented with 4-aminopyridine (4AP), and control Ames medium again (to check for reversibility). All solutions were bubbled with carbogen, warmed by a heater beneath the recording chamber, and flowed through the recording chamber at ∼2 mL/min. The superfusate temperature was maintained at a constant temperature that was above 30°C and as close to 37°C as stable recordings could be made. The stimulus pulses were delivered once per 5 s from an optically isolated stimulator (STG 4002; Multi Channel Systems) that was controlled by a PC computer program (MC_Stimulus II, version 3.0.1; Multi Channel Systems, RRID:SCR_014955). The stimuli consisted of 100-μs biphasic pulses of ± 1–16 V (50:50 duty cycle, negative first). Spikes were recorded with MEA electrodes (60MEA200/30iR-ITO, MultiChannel Systems), bandpass filtered (1 Hz to 3 kHz, 60 dB/decade), and sampled at 25 kHz/channel using pCLAMP (version 9.2.1.9; Molecular Devices, RRID:SCR_011323).

MC_Rack (Multi Channel Systems, RRID:SCR_014955), MC_DataTool (Multi Channel Systems, RRID:SCR_014580), Clampfit (version 10; Molecular Devices, RRID:SCR_011323), and Excel (Microsoft, RRID:SCR_016137) were used to analyze the electrophysiological recordings. Two-tailed paired Student’s *t*-tests, one-way ANOVA, two-way ANOVA, and Tukey’s multiple comparisons tests were performed using Prism9 (GraphPad, RRID:SCR_002798). Unless stated otherwise, measurements are presented as the mean ± standard error of the mean (S.E.M.), plus sample size.

### Fixation and immunostaining

For immunostaining, optic nerves were dissected from the back of the eye to the proximal side of the optic foramen, immersed overnight at 4°C in a fixative containing formaldehyde (4% w/v) in phosphate-buffered saline (PBS; pH 7.4), embedded in 5% (w/v) low-melt agarose at 37°C, allowed to set for at least 60 min at 4°C, cut into blocks with a razor blade, and sectioned longitudinally at a thickness of 75−100 μm on a vibrating microtome (VT1000S; Leica Microsystems). Free-floating sections were blocked and permeabilized for 24 h at 4°C in TBST (Tris-buffered saline, 0.3% v/v Triton-X100, pH 7.4) that was supplemented with 5% normal donkey serum. The sections were incubated in primary antibody (either overnight at 37°C or for as long as 5 days at 4°C), rinsed with PBS, incubated in secondary antibody (overnight at either 37°C or 4°C), rinsed with PBS, and mounted in Prolong Diamond Antifade Mountant between glass coverslips (No. 1.5) and glass slides. Primary and secondary antibodies were diluted in TBST.

### Confocal imaging and image handling

Confocal images were acquired on a Leica TCS SP8 STED 3X high-sensitivity confocal microscope, coupled with Leica Application Suite X software (Leica, RRID:SCR_013673). The results reported here were collected by use of excitation lines for AF488 (499 nm), Cy3 (554 nm), and AF647 (653 nm), an oil immersion 63X objective (HC PL APO CS2, numerical aperture = 1.4), optical sections at step sizes of 0.180 μm, and five-frame Kalman averaging for each optical section. The voxel size was 180 × 180 × 180 nm. For most imaging sessions, some sections were processed with primary and secondary antibodies, some sections were processed without primary antibody (i.e., with secondary antibody alone), and the laser intensity, pinhole diameter, and photomultiplier gain were selected so that the secondary antibody fluorescence intensity was indistinguishable from background fluorescence. To test for non-selective binding of primary antibodies, some sections were processed with primary antibody that was preincubated with immunogen at the concentration recommended by the manufacturer (see below).

Data sets were imported into the ImageJ package Fiji.^1^ Changes in color space, adjustments to brightness or contrast (if any), and image overlays were made in Photoshop CS6 (Adobe Systems, RRID:SCR_014199).

### Protein isolation

For western blotting, optic nerves were dissected from the back of the eye to the proximal side of the optic foramen, placed in a capped microcentrifuge tube, frozen by dropping into liquid nitrogen, and homogenized in homogenization buffer (250 mM sucrose, 250 mM Na_3_VO_4_, 25 mM NaF, 1 mM MgCl_2_, 1 mM dithiothreitol, 20 mM Tris-HCl, pH 7.5). The homogenates were centrifuged at 600g for 10 min at 4°C. The resulting pellets were discarded, and the supernatant was concentrated in centrifugal filters (Amicon 30 kD, EMD Millipore). The concentrated homogenate was diluted in 200 μL of dilution buffer (320 mM sucrose, 50 mM MgCl_2_, 100 mM Tris-HCl; pH 7.4), concentrated again, and then diluted in a final volume of 65 μL of dilution buffer. The homogenization and dilution buffers were ice-cold and supplemented with protease inhibitor (cOmplete Mini, Roche) and phosphatase inhibitor (PhosSTOP, Roche) tablets. This report refers to the diluted homogenate as lysate. The total protein concentration of the lysate was determined using a bicinchoninic acid protein assay kit.

### Immunoprecipitation

For protein samples used in immunoprecipitation experiments, optic nerve lysate was pre-cleared by incubating on a rocker at 4°C for 4 h with protein A magnetic beads (EMD Millipore). Pre-cleared lysate was then immunoprecipitated with either rabbit polyclonal anti-Kv4.3 antibody or mouse monoclonal anti-CaMKII antibody, using a reversible immunoprecipitation system (Catch and Release v2.0, Cat # 17-500A, EMD Millipore). Immunoprecipitated proteins were eluted in a non-denatured form and prepared for Western blotting as described below.

### Western blot

Aliquots of optic nerve lysate and immunoprecipitated proteins were loaded into 4–12% gradient gel lanes (Bis-Tris, Invitrogen), electrophoretically separated in MOPS running buffer at 200 V for 1 h, and transferred to polyvinyl difluoride (PVDF) membranes (Bio-Rad) at 30 V for 1 h. Protein standards (SeeBlue Plus2) were run in lanes adjacent to the samples. Each PVDF membrane was immersed for 1 h in a protein-supplemented buffer solution (Superblock), and then incubated with primary antibodies on a rocker overnight at 4°C. As a loading control, lanes were also immunostained for either β-actin or myelin basic protein. The membranes were then rinsed with Tris-buffered saline (supplemented with 0.5% Tween 20) and incubated in species-specific, fluorophore-conjugated secondary antibodies for 1 h at room temperature. The fluorescence of these fluorophores was visualized and imaged in a digital imager (FluorChem Q, Alpha Innotech; RRID:SCR_014549). The molecular weights of the immunostained protein bands were analyzed by use of the imager software (AlphaView Q, ProteinSimple). Unless stated otherwise, measurements are presented as the mean ± S.E.M., plus sample size.

### Antibody characterization

The rabbit polyclonal anti-Kv4.3 antibody (Alomone, RRID:AB_2040178) was generated against amino acids 451–468 of human Kv4.3. It was used on Western blots and for immunostaining at a dilution of 1:400.

The Kv4.3 blocking peptide (Alomone, BLP-PC017) is a synthetic peptide corresponding to amino acid residues 451–468 of human Kv4.3. It was used at the concentration recommended by the manufacturer (1 μg peptide per 1 μg antibody).

The following antibodies were used on Western blots only (at a dilution of 1:400, except where noted): mouse monoclonal anti-CaMKII antibody (Santa Cruz Biotechnology, clone G-1, RRID:AB_626788) generated against amino acids 303-478 of mouse CaMKIIα; rabbit polyclonal anti-Kv4.3 antibody (Thermo Fisher, RRID:AB_2576642) generated against the middle region of human KCND3; mouse monoclonal anti-Kv4.3 antibody (Sigma-Aldrich, RRID:AB_2783025) generated against amino acids 415–636 (cytoplasmic C-terminus) of rat Kv4.3; goat polyclonal anti-β-actin antibody (Abcam, RRID:AB_306374) generated against a synthetic peptide (within human β-actin amino acids 1–100) that was conjugated to keyhole limpet hemocyanin; and rat monoclonal anti-myelin basic protein (MBP) IgG2a antibody (Abcam, clone 12, RRID:AB_305869) that was generated against full length cow MBP, binds to a region defined by amino acids 82–87, and used at a dilution of 1:1000.

The following antibodies were used for immunostaining only (all at a dilution of 1:400): rabbit polyclonal anti-Kv4.2 antibody (Alomone, RRID:AB_2040176) generated against amino acids 454–469 of rat Kv4.2; mouse monoclonal IgG_1_ anti-Kv4.2 antibody (NeuroMab, RRID:AB_2877281) generated against a synthetic peptide corresponding to amino acids 209–225 of rat Kv4.2; mouse monoclonal IgG_1_ anti-Kv4.2 antibody (NeuroMab, RRID:AB_2877425) generated against a fusion protein corresponding to amino acids 471-630 of rat Kv4.2; rabbit polyclonal anti-Kv1.4 antibody (Alomone, RRID:AB_2040153) generated against a GST fusion protein corresponding to amino acid 589-655 of rat Kv1.4; mouse monoclonal IgG_1_ anti-Kv1.4 antibody (NeuroMab, RRID:AB_2877317) generated against a synthetic peptide corresponding to amino acids 13–37 of rat brain Kv1.4; mouse monoclonal IgG_1_ anti-Kv1.4 antibody (NeuroMab, RRID:AB_2877393) generated against a fusion peptide of amino acids 336-370 of rat Kv1.4; and mouse monoclonal IgG_1_ anti-NF-L antibody (Millipore MAB1615) directed against enzymatically dephosphorylated pig neurofilament-L (70 kDa).

Signals attributed to the primary antibodies were visualized with species-specific, fluorophore-conjugated, donkey or mouse secondary antibodies (Jackson ImmunoResearch Laboratories, RRID:AB_2340684, AB_2340863, AB_2339171, AB_2492288). The secondary antibodies were typically diluted to 1:500 for immunostaining and to 1:1000 or 1:2000 for Western blotting. Secondary antibodies used for some of the Western blots were light chain specific. The fluorophores were AlexaFluor 488, Cy3, or AlexaFluor 647.

### Reagents

Reagents were obtained from the following sources: Bio-Rad [Tween 20, Tris-buffered saline (pH 7.4)]; Jackson ImmunoResearch (normal donkey serum); Life Technologies (ProLong Diamond Antifade Mountant); Roche (cOmplete Mini, PhosSTOP); Sigma-Aldrich (4-aminopyridine, ASB-14, dithiothreitol, ethanol, formaldehyde, MgCl_2_, NaF, Na_3_VO_4_, sucrose); Thermo Fisher Scientific (bicinchoninic acid protein assay kit, low melting point agarose, MOPS running buffer, Restore PLUS Western Blot Stripping Buffer, SeeBlue Plus2 Pre-Stained Protein Standard, SuperBlock, Triton X-100); United States Biologicals [buffered Ames medium (A1372-25)]; and Western Medical Supply (ketamine, xylazine).

## Results

The proximate goal of this study is to identify 4-aminopyridine (4AP)-sensitive K^+^ current that CaMKII might modulate in order to alter spike propagation speed in rat optic nerve. Given the age-dependence of K^+^ efflux from optic nerve (Connors et al., 1982) and of the degree that 4AP increases the duration of optic nerve compound action potentials (Devaux et al., 2002), we used adult rats (P60-P120) for all of our experiments. We first tested whether 4AP alters spike shape and slows spike propagation. We next asked whether optic nerve binds antibodies directed against subtypes of K^+^ channel that are regulated by CaMKII and blocked by 4AP. Lastly, we tested whether CaMKII co-immunoprecipitates with the 4AP-sensitive K^+^ channel subunit we detect in optic nerve.

### 4AP slows spike repolarization

The ability of 4AP to slow spike repolarization (i.e., to slow the return of membrane potential toward resting potential) is commonly attributed to reducing outward K^+^ current (Storm, 1987). Because we used multi-electrode array electrodes to record axonal spikes, we could detect changes in the rate of post-peak repolarization by changes in the amplitude of P3 (the upward peak following the downward peak) of the triphasic spike waveforms (see Materials and methods; Meister et al., 1994; Henze et al., 2000; Fogli Iseppe et al., 2020). In recordings at one electrode (Figure 1A), the amplitude of P3, averaged across 10 spikes recorded in each solution, was 123 ± 4 μV in control solution, 16 ± 4 μV in 4AP-containing solution, and 55 ± 5 μV after the preparation was washed with control solution (Figure 1B, left). The mean of these amplitudes in 4AP-containing solution was 13% of that in control solution, and the difference between these amplitudes was statistically significant (one-way ANOVA and Tukey’s multiple comparisons test, *p* < 0.0001, *n* = 10). Similar results were obtained in 2 other preparations. Averaged across the spikes recorded at a total of 9 electrodes in a total of 3 preparations (Figure 1C, left), 4AP decreased the amplitude of P3 to 45 ± 13% of the control value (two-way ANOVA and Tukey’s multiple comparisons test, *p* < 0.0001, *n* = 30).

As would be expected if 4AP selectively slows spike repolarization, 4AP reduced the amplitude of P3 without markedly changing the amplitude of P2 (the downward peak that immediately preceded each P3). For the traces illustrated in Figure 1A, the mean of the amplitudes of P2 in 4AP-containing solution was 102% of that in control solution, and the difference between these amplitudes was not statistically significant (one-way ANOVA and Tukey’s multiple comparisons test, *p* = 0.605, *n* = 10; Figure 1B, middle). This indicates that 4AP did not significantly change the maximum rate of membrane potential change as the spikes depolarized toward their peaks (Meeks et al., 2005; Fogli Iseppe et al., 2020). Similar results were obtained in 2 other preparations. Averaged across the spikes recorded at a total of 9 electrodes in a total of 3 preparations (Figure 1C, middle), the amplitude of P2 in 4AP-containing solution was 100 ± 3% of the control value (two-way ANOVA and Tukey’s multiple comparisons test, *p* = 0.4261, *n* = 30).

Because 4AP selectively slowed spike repolarization, spike duration increased. We estimated this increase from the times that elapsed between P2 and P3 of each triphasic spike, because we previously found that these elapsed times are proportional to intracellularly recorded spike duration (Fogli Iseppe et al., 2020). Averaged across 10 spikes recorded in each solution, these times in Figure 1A were 0.4 ± 0.01 msec in control solution, 1.2 ± 0.03 msec in 4AP-containing solution, and 1.2 ± 0.03 msec when the preparation was washed with control solution (Figure 1B, right). The average of the means of these elapsed times was 194% longer in 4AP-containing solution than that in control solution, and the difference between these times was statistically significant (one-way ANOVA and Tukey’s multiple comparisons test, *p* < 0.0001, *n* = 10). Averaged across the spikes analyzed above for P3 amplitude (Figure 1C, right), the elapsed times were 0.4 ± 0.1 msec in control solution and 1 ± 0.1 msec in 4AP-containing solution. The difference between these times was statistically significant (two-way ANOVA and Tukey’s multiple comparisons test, *p* < 0.0001, *n* = 30), with 4AP increasing the P2-to-P3 elapsed times by 215 ± 27% over the control value.

These results are consistent with 4AP reducing outward K^+^ current that repolarizes spikes (Storm, 1987; Liu et al., 2017) and, in turn, support the immunostainings and immunoprecipitations presented below to identify a 4AP-sensitive K^+^ channel in optic nerve.

### 4AP slows spike propagation

Given that KN-93, okadaic acid, and fostriecin alter the time-to-peak of optic nerve compound action potentials (Partida et al., 2018), and given our goal of identifying a K^+^ current that CaMKII might modulate in order to alter spike propagation, we next tested whether 4AP alters spike propagation and reduces the amplitude of P3. In these recordings, the stimulating electrode was placed at the cut end of an optic nerve and the recording electrode was in a multi-electrode array on which the attached retina was laid ganglion cell-side down (see Materials and methods). Addition of 4AP (300 μM) to the solution superfusing the preparation increased the time that elapsed between the stimulus artifact and P1 (the first peak of the triphasic spike waveform, at the dotted vertical lines in Figures 2A,C). This is consistent with a decrease in propagation speed (cf., Wässle et al., 1975). In Figure 2A, the stimulus-to-P1 time measured 3.6 ± 0.2 msec in control solution and 4.3 ± 0.1 msec in 4AP-containing solution (3 spikes recorded in each solution). The mean of these times was 19% longer in 4AP-containing solution than in control solution, and the difference between these times was statistically significant (two-tailed, paired Student’s *t*-test, *p* = 0.0267, *n* = 3). Similar results were obtained in 2 other preparations. The means of the stimulus-to-P1 times in these preparations were longer (16 ± 0.3% and 19 ± 4%, respectively) in 4AP-containing solution than in control solution. In the 3 preparations we recorded from, the difference between the stimulus-to-P1 times in control and 4AP-containing solution was statistically significant (two-tailed, paired Student’s *t*-test, *p* < 0.0001, *n* = 10; Figure 2D). Moreover, the three rows of spikes in Figure 2 show that 4AP slowed propagation and reduced the amplitude of P3 without consistently or markedly altering the amplitude of P2. Together, the effect of 4AP on spike propagation and repolarization would increase time-to-peak, and broaden peaks, of compound action potentials, as found in responses to repetitive spiking and to KN-93 (Partida et al., 2018).

The 4AP concentrations we used (0.3−1.0 mM) are within the range of concentrations used in studies of optic nerve compound action potentials (Foster et al., 1982; Rasband et al., 1999; Brown et al., 2001; Devaux et al., 2002). Those studies, and ours, did not test for effects of saturating concentrations of 4AP (e.g., Serôdio et al., 1996). Also, we know of no K^+^ channel antagonist that is specific for the Kv4.3 isoform that we focus on below (Sanguinetti et al., 1997; Sergeant et al., 2005; Diochot et al., 2009; Jeong et al., 2011, 2012; Fischer et al., 2013; Maffie et al., 2013; Kimm and Bean, 2014; see also Grissmer et al., 1994; Mlayah-Bellalouna et al., 2014; Haddjeri-Hopkins et al., 2021) and therefore did not test for effects of antagonists beside 4AP (e.g., heteropodatoxin, flecainide, phrixotoxin, dapoxetine, duloxetine, rosiglitazone, ajmaline, AmmTX3, SNX-482).

### Anti-Kv4.3 antibodies bind to optic nerve axons

**FIGURE 3:**
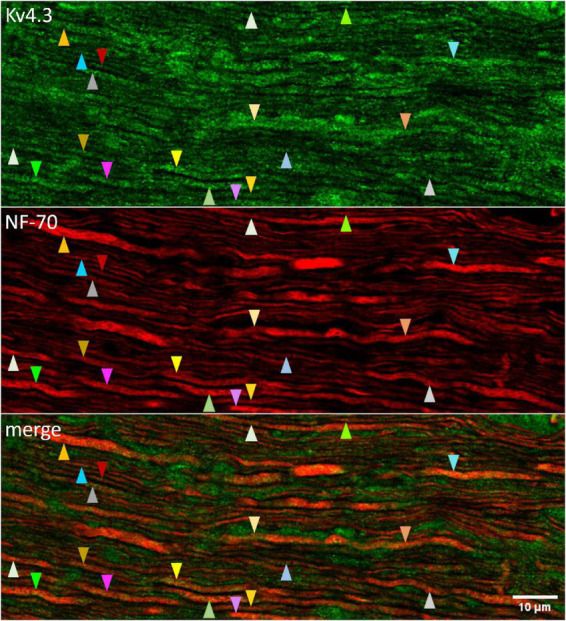
Kv4.3 immunopositivity in optic nerve axons. Confocally imaged portion of optic nerve that was longitudinally sectioned and immunostained with antibodies directed against Kv4.3 (upper panel) and NF-70 (middle panel). The upper and middle panels are superimposed in the lower panel (“merge”). The tip of each arrowhead points to an axon (based on NF-70 immunopositivity). Arrowheads of a given color point at the same axon. Different colors are used to point at different axons. Otherwise, the color and direction of the arrowheads (upward or downward) are arbitrary. The calibration bar in the lower panel shows 10 μm and applies to all panels.

**FIGURE 4:**
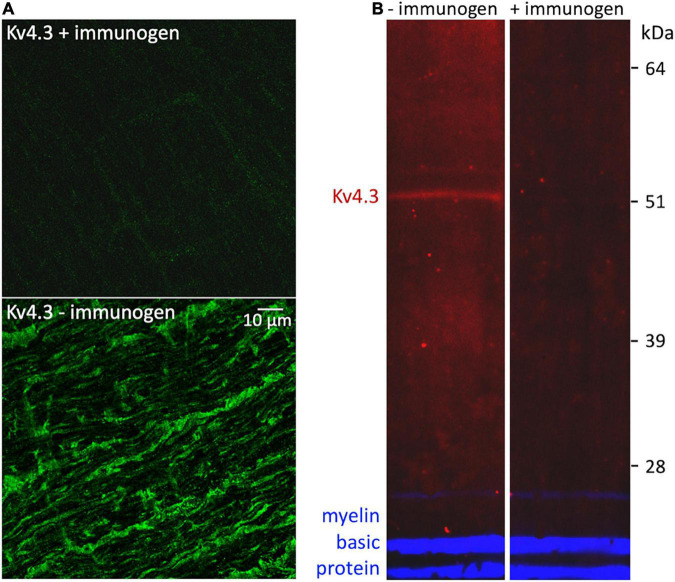
Control for non-specific binding by rabbit anti-Kv4.3 antibody. **(A)** Fields of optic nerve that were longitudinally sectioned and processed as in Figure 3, except that the anti-Kv4.3 antibody in the upper panel was preincubated with immunogen, and the anti-Kv4.3 antibody in the lower panel was not (as in Figure 3). These fields were imaged at identical confocal microscope settings (laser intensity, photomultiplier gain, and pinhole diameter). The calibration bar in the lower panel shows 10 μm and applies to both panels. **(B)** Western-blotted lanes of optic nerve lysate. The lanes were processed identically except that the anti-Kv4.3 antibody was either preincubated with immunogen (in the lane marked “+immunogen”) or it was used without this preincubation (in the lane marked “–immunogen”). To gauge protein load, both lanes were probed with rat anti-myelin basic protein antibody. The fluorescence of the anti-rabbit secondary antibody is pseudo-colored red. The fluorescence of the anti-rat secondary antibody is pseudo-colored blue. The molecular weight and migration distance of protein standards that were run in an adjacent lane are shown along the right edge.

4AP impedes, and KN-93 modulates, current flow through K^+^ channels formed by the subunit isoforms Kv1.4, Kv4.2, and Kv4.3 (Roeper et al., 1997; Koh et al., 1999; Colinas et al., 2006). To identify 4AP-sensitive K^+^ channels that CaMKII might associate with in optic nerve, we used standard indirect immunofluorescence immunostaining methods to test whether antibodies directed against those isoforms bind to sections of optic nerve. We found that an anti-Kv4.3 antibody (Alomone, RRID:AB_2040178) bound to fiber-shaped profiles in optic nerve (Figure 3, upper and lower panels). We identified those structures as axons because they also bound antibody directed against NF-70 (Figure 3, middle and lower panels), a neurofilament found in axons (Van der Zee et al., 1997). The arrowheads in Figure 3 point at segments of Kv4.3-immunopositive axons (*n* = 19). These range in diameter between 0.3 and 1.9 μm, and extend for continuous lengths as long as 50 μm. These segments thus resembled the small, medium, and large-diameter axons that we previously found to bind antibodies directed against pan axonal neurofilaments (SMI-312; Partida et al., 2018). We did not notice a caliber of axon that consistently lacked Kv4.3-immunopositivity (Figure 3), and no Kv4.3 immunostaining above background was detected when the primary antibody was preincubated with immunogen (Figure 4A).

By contrast, we did not detect binding of either anti-Kv1.4 antibodies (Alomone, RRID:AB_2040153; NeuroMab, RRID:AB_2877317, RRID:AB_2877393) or anti-Kv4.2 antibodies (Alomone, RRID:AB_2040176; NeuroMab RRID:AB_2877281, RRID:AB_2877425) to optic nerve (images not illustrated).

### Calcium/calmodulin-dependent protein kinase II co-immunoprecipitates with Kv4.3

**FIGURE 5:**
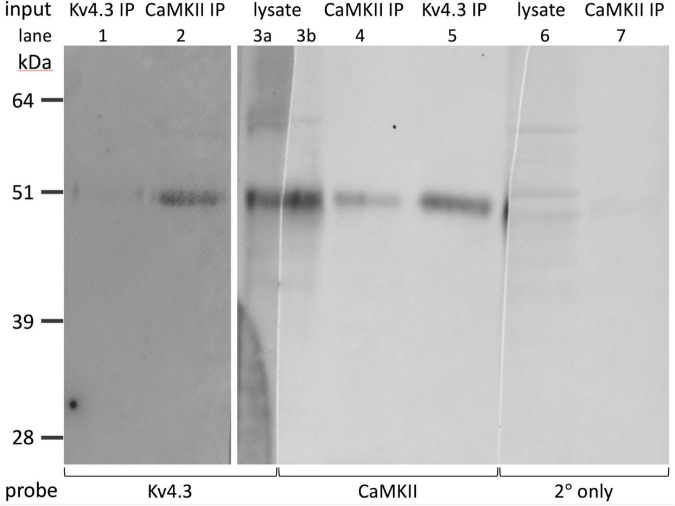
Reciprocal co-immunoprecipitation of Kv4.3 and CaMKII. Western-blotted lanes (1-7), with one lane cut vertically into two parts (lanes 3a, 3b). The material that was loaded into the gel lanes for electrophoresis, and the antibody used to probe each Western-blotted lane, are listed above and below the lanes, respectively. The lanes were loaded with either optic nerve lysate (lanes 3a, 3b, 6), elute from the anti-Kv4.3 antibody pull-down (lanes 1, 5), or elute from the anti-CaMKII antibody pull-down (lanes 2, 4, 7). The Western-blotted lanes were probed with rabbit anti-Kv4.3 antibody (“Kv4.3”) and anti-rabbit secondary antibody, mouse anti-CaMKII antibody (“CaMKII”) and anti-mouse secondary antibody, or anti-rabbit secondary antibody without primary antibody (“2° only”). The molecular weight and migration distance of protein standards that were run in an adjacent lane are shown along the left edge. Note that the faint bands around 60 kDa in lane 3a are absent in lane 3b.

The localization of Kv4.3 and CaMKII in optic nerve axons described and cited above raises the possibility that Kv4.3 and CaMKII associate with each other. We tested this possibility by co-immunoprecipitation and by reciprocal co-immunoprecipitation (Figure 5), using the anti-Kv4.3 antibody we used in our immunostainings (Figure 3) and anti-CaMKII antibody instead of anti-pT286 antibody (Colinas et al., 2006; Keskanokwong et al., 2011).

The Kv4.3 antibody (Alomone, RRID:AB_2040178) immunoprecipitated protein that was detectable by anti-Kv4.3 antibodies directed against three different epitopes (Alomone, RRID:AB_2040178; Thermo Fisher, RRID:AB_2576642; Sigma-Aldrich, RRID:AB_2783025) and co-immunoprecipitated protein that was detectable by anti-CaMKII antibody (Santa Cruz, RRID:AB626788). In the 7 pairs of Western blot lanes we prepared and immunostained in parallel, the migration distance of the immunoprecipitated Kv4.3-immunopositive bands hardly differed from the migration distance of the co-immunoprecipitated CaMKII-immunopositive bands. The apparent molecular weights estimated from these distances were 51 ± 0.3 kDa for Kv4.3 (*n* = 7) and 52 ± 0.4 kDa for CaMKII (*n* = 7). The differences between these values were not statistically significant (2-tailed, paired Student’s *t*-test, *p* = 0.18). In 2 Kv4.3-immunostained lanes, a slightly higher molecular weight band was also stained (53 kDa in one, 55 kDa in the other). A slightly higher molecular weight band was also found in the CaMKII-immunostained lanes run parallel to these (53 kDa in both cases).

Reciprocal co-immunoprecipitations were run side-by-side, using anti-CaMKII and anti-Kv4.3 antibodies to immunoprecipitate proteins and, after Western blotting, to probe for immunoprecipitated and co-immunoprecipitated proteins. The anti-CaMKII antibody immunoprecipitated protein that was detectable by anti-CaMKII antibody (Figure 5, lane 4), co-immunoprecipitated protein that was detectable by anti-Kv4.3 antibody (Figure 5, lane 2), and bound to protein in the elute from the anti-Kv4.3 antibody pull-down (Figure 5, lane 5). The anti-Kv4.3 antibody immunoprecipitated protein that was detectable by anti-Kv4.3 antibody (Figure 5, lane 1; see below), co-immunoprecipitated protein that was detectable by anti-CaMKII antibody (Figure 5, lane 5), and bound to protein in the elute from the anti-CaMKII antibody pull-down (Figure 5, lane 2). Averaged across the 2 reciprocal co-immunoprecipitations we ran, the estimated apparent molecular weight of the CaMKII-immunopositive bands that were co-immunoprecipitated by the anti-Kv4.3 antibody was 50 kDa, and the estimated apparent molecular weight of the Kv4.3-immunopositive bands that were co-immunoprecipitated by the anti-CaMKII antibody was 51 kDa.

Averaged across all of the CaMKII-immunoprecipitated, Kv4.3 co-immunoprecipitated, and optic nerve lysate (e.g., Figure 5, lane 3b) lanes we probed, the estimated apparent molecular weight of the CaMKII-immunopositive bands was 51 ± 0.3 kDa (*n* = 19). This is consistent with estimated apparent molecular weights reported for CaMKII by other studies (Ochiishi et al., 1994; Laabich and Cooper, 1999). Averaged across all of the Kv4.3-immunoprecipitated, CaMKII-co-immunoprecipitated, and optic nerve lysate lanes we probed, the estimated apparent molecular weight of the Kv4.3-immunopositive bands was 52 ± 0.3 kDa (*n* = 24). These averages do not include estimated apparent molecular weights of the higher molecular weight bands, like those mentioned above, because they were not consistently seen. However, these bands are notable because they were occasionally stained by either the anti-Kv4.3 antibody or the anti-CaMKII antibody, but not by both (e.g., Figures 5A,B). Preincubating the anti-Kv4.3 antibody with immunogen reduced the immunostaining of optic nerve lysate (Figure 4B, left lane) to undetectably low levels (Figure 4B, right lane). Also, we detected no binding of the secondary antibody alone to protein immunoprecipitated by the anti-CaMKII antibody (Figure 5, lane 7). These controls show that there was no detectable non-specific binding of the anti-Kv4.3 antibody or of the secondary antibody used to visualize it. In particular, the latter indicates that the binding of anti-Kv4.3 antibody to the ∼50 kDa band in lanes 2 and 3a of Figure 5 is not due to binding of the secondary antibody to CaMKII.

The anti-Kv4.3 antibody staining of elutes from anti-Kv4.3 antibody pull-downs was faint (e.g., Figure 5, lane 1). However, we obtained similar results with antibodies directed against different Kv4.3 epitopes (see above). Also, anti-Kv4.3 antibodies have been found to stain protein bands at higher molecular weight in some studies (Rhodes et al., 2004; Colinas et al., 2006; Norris and Nerbonne, 2010). However, the estimated apparent molecular weight in our blots, and the reduction of immunostaining intensity by preincubating the primary antibody with immunogen, agree with findings of other studies (Zicha et al., 2004; Jin et al., 2010). Altogether, the above results show that CaMKII and Kv4.3 co-immunoprecipitate with each other, and are consistent with the possibility that CaMKII and Kv4.3 associate with each other in optic nerve.

## Discussion

The primary findings of this study are that 4AP slows axonal spike propagation, that Kv4.3 immunopositivity is present in optic nerve axons, and that optic nerve CaMKII and Kv4.3 co-immunoprecipitate with each other. Below, we compare these results to K^+^ channel subunit localizations reported for other neurons and discuss how our results might account for CaMKII regulation of spike propagation speed in optic nerve.

### Optic nerve Kv4.3

K^+^ channel subunit immunostainings in rat optic nerve differ from those in other preparations in two respects. One is that Kv1.4 is not detected in rat optic nerve (Rasband et al., 1999), but is concentrated in axons in the hippocampal mossy fiber pathway and basal ganglia (Sheng et al., 1992; Rhodes et al., 1997). A second difference is our finding of Kv4.3 in adult rat optic nerve (Figures 3-5) versus its presence in dendrites and/or somata of several cell types in hippocampus, striatum, cingulate cortex, visual cortex, hypothalamus, and spinal cord (Rhodes et al., 2004; Huang et al., 2005; Strassle et al., 2005; Burkhalter et al., 2006; Chien et al., 2007; Arroyo et al., 2011). We do not yet know why K^+^ channel subunit immunostainings in optic nerve and these other areas would differ. Could the presence of Kv4.3 confer a functional advantage on optic nerve? An earlier study found Kv4.3 immunopositivity in developing *Xenopus* retinal ganglion cell axons and that 4AP disrupted their extension and projections (McFarlane and Pollock, 2000). A subsequent study of rat optic nerve reported that Kv4.3 immunopositivity is detectable in late embryos and absent in early postnates, but did not list results for adults (Huang et al., 2012). Apart from the results presented here, we are aware of only one other study that found Kv4.3 in adult axons (specifically, in carotid sensory nerve fibers; Buniel et al., 2008). Kv4.3 presence in adult axons therefore appears to be rare.

One difference between the protocols and materials in our experiments versus those of studies that found Kv4.3 in somato-dendritic compartments (e.g., Huang et al., 2005; Chien et al., 2007; Arroyo et al., 2011) is the rat strain used (Long-Evans versus Sprague-Dawley). However, the previous finding of Kv4.3 in axons was made in adult Sprague-Dawley rats (Buniel et al., 2008), implying that differences in rat strain do not account for our having found Kv4.3 in optic nerve. Furthermore, other studies that found Kv4.3 in somato-dendritic compartments of Sprague-Dawley rats (Rhodes et al., 2004; Strassle et al., 2005) used different primary antibodies than our study and the studies by Huang, Chien, Arroyo and colleagues (*ibid*.). This implies that differences in primary antibody do not account for our having found Kv4.3 in optic nerve. Based on these results, plus the ability of anti-CaMKII antibody to co-immunoprecipitate Kv4.3 from Long-Evans rat optic nerve (Figure 5), and similarities between the effect of 4AP on optic nerve compound action potentials in Lewis, Wistar, and Long-Evans rats (Gordon et al., 1988; Rasband et al., 1999; Brown et al., 2001; Devaux et al., 2002), we did not test whether our protocols would show Kv4.3 immunopositivity in Sprague-Dawley rat optic nerve.

Our results indicate that Kv4.3 is present in adult rat optic nerve axons. Moreover, we did not detect Kv4.2 or Kv1.4 in adult rat optic nerve. These results are consistent with the finding of Kv4.3, without Kv4.2, in hippocampal, striatal, and neocortical interneurons, substantia nigra dopaminergic neurons, medial preoptic area GnRH neurons, and dorsal root ganglion nocioceptive neurons (Liss et al., 2001; Rhodes et al., 2004; Phuket and Covarrubias, 2009; Arroyo et al., 2011) and, as noted above, the finding by Rasband et al. (1999) that Kv1.4 is absent in rat optic nerve. At the same time, our results do not exclude the possibility that Kv4.2 is present in a small number of rat retinal ganglion cells, as found in mice (Qu et al., 2009). The possibility thus remains that Kv4.3 and Kv4.2 are co-expressed in some retinal ganglion cells and/or are expressed in different retinal ganglion cells (cf., Rhodes et al., 2004; Burkhalter et al., 2006), and that modulation of Kv4.2 by CaMKII (Varga et al., 2004) might also contribute to modulation of optic nerve spike propagation.

### Optic nerve Kv4.3 and calcium/calmodulin-dependent protein kinase II

Spikes activate voltage-gated Ca^2+^ currents and, in turn, raise intracellular free Ca^2+^ concentration in adult retinal ganglion cell axons (Ren et al., 2000; Behrend et al., 2009). The observation that retinal ganglion cells can generate spikes even after Ca^2+^ currents are blocked (Murakami and Shimoda, 1977; Margolis and Detwiler, 2007) implies that Ca^2+^ currents are not required for spike generation, but might instead equip retinal ganglion cells to regulate spikes. Possible effectors of this regulation have been found, including protein kinase C (Komoly et al., 1991), calmodulin (Brady et al., 1981), CaMKII (Lund and McQuarrie, 2001; Calkins et al., 2005), calcineurin (Seitz et al., 2002), and protein phosphatase 2A (PP2A; Partida et al., 2018). We previously found that the time-to-peak of optic nerve compound action potentials can be modulated by reagents that alter pT286 levels (Partida et al., 2018). We have extended those results here by identifying Kv4.3 as a K^+^ channel isoform that associates with CaMKII. This is a novel find in optic nerve and it is consistent with reports that cardiac myocyte Kv4.3 and CaMKII co-immunoprecipitate (Colinas et al., 2006; El-Haou et al., 2009) and that pT286 modulates K^+^ current passing through channels formed by Kv4.3 subunits (Sergeant et al., 2005).

Kv4.3 appears to be appropriate for regulating spike propagation in multiple respects. Firstly, we found Kv4.3 immunopositivity continuously along lengths of optic nerve axons for several micrometers (e.g., as long as ∼50 μm in Figure 3). It is thus distributed like activity-induced increases in intra-axonal free Ca^2+^ concentration (Zhang et al., 2006) and immunopositivities for pT286 (Partida et al., 2018), PP2A (Partida et al., 2018), and voltage-gated Ca^2+^ channels (Brown et al., 2001), as would be expected if Ca^2+^ influx sites, CaMKII, PP2A, and Kv4.3 channels were positioned near each other and thereby facilitate their interaction. These overlapping distributions might reflect, or allow for, association of CaMKII, PP2A, and/or voltage-gated Ca^2+^ channels, like that found here between CaMKII and Kv4.3 (Figure 5), and those found in previous studies between CaMKII and voltage-gated Na^+^, Ca^2+^, Cl^–^, and other K^+^ channels (Sun et al., 2004; Hudmon et al., 2005; Wagner et al., 2006; Yao et al., 2006; Cuddapah and Sontheimer, 2010), between CaMKII and PP2A (Zhang et al., 2002; Yamashita et al., 2006; Lu et al., 2009), and between PP2A and other voltage-gated ion channels (Davare et al., 2000; El Refaey et al., 2019). Secondly, the voltage-sensitivity of Kv4.3 channel activation and steady-state inactivation indicate that Kv4.3 channels can contribute to membrane resistance at membrane potentials near to, and more positive than, resting potential (Sergeant et al., 2005; Colinas et al., 2006; Qu et al., 2007). Consistent with 4AP-sensitive channels contributing to this membrane resistance, 4AP enables anode break to elicit spikes in adult rat optic nerve (Eng et al., 1990; cf., Debanne et al., 1997). Moreover, KN-93 would be expected to increase this resistance, because it reduces current flow through Kv4.3 channels by (*i*) reducing the maximum current that can be activated, (*ii*) reducing the current activated by moderate depolarizations (i.e., shifting the activation curve to more depolarized membrane potentials), (*iii*) speeding the loss of current during depolarizations (i.e., accelerating the rate of open-state inactivation), and (*iv*) slowing the rate of recovery from inactivation, all without changing the voltage-sensitivity of steady-state inactivation (Qu et al., 2007). Importantly, these effects do not occur if the S516 and S550 CaMKII consensus phosphorylation sites in Kv4.3 are mutated (Qu et al., 2007). Previously derived equations (Jack et al., 1975; Matsumoto and Tasaki, 1977; Walton and Fozzard, 1983) predict that, by increasing membrane resistance formed by Kv4.3 channels, KN-93 and activity-induced decreases in pT286 will slow spike propagation. Thus, the results of the present study, and of studies cited above, appear to be consistent with events in the following sequence:

1) optic nerve spikes activate voltage-gated Ca^2+^ channels in retinal ganglion cell axons;
2) Ca^2+^ influx through these channels increases intra-axonal free Ca^2+^ concentration;
3) increases in Ca^2+^ concentration lower intra-axonal concentration of pT286;
4) decreased level of activated CaMKII decreases conductance formed in retinal ganglion cell axons by Kv4.3 (by reducing activation, and increasing inactivation, of channels formed by Kv4.3);
5) decrease in conductance formed by Kv4.3 increases membrane resistance;
6) increase in membrane resistance slows the propagation of spikes that fire after the spikes that reduced intra-axonal pT286.

The molecular composition of ion channels formed in retinal ganglion cell axons by Kv4.3, and the phosphatases that reduce CaMKII activation, remain to be identified (cf., Colbran, 2004; Wen et al., 2004; Bayer and Shulman, 2019). Also, whether the changes in phosphorylation entailed can occur during the shortest interspike intervals known to slow spike propagation remains to be tested.

### Na^+^ and other K^+^ conductances

Our results do not exclude the possibility that activity slows spike propagation by other effects on ion currents. For example, reduction of voltage-gated Na^+^ current by tetrodotoxin (Colquhoun and Ritchie, 1972) and by QX-314 (Hessler et al., 1993) have been found to slow spike propagation, and voltage-induced inactivation of voltage-gated Na^+^ current in unmyelinated axons is thought to slow spike propagation (De Col et al., 2008). We have not tested for any of these effects in optic nerve. However, we have compared spikes that fired in pairs, in either optic chiasm or optic tract. We found no amplitude differences in these spikes (Fogli Iseppe et al., 2020), even when they fired at interspike intervals that inactivate Na^+^ current in retinal ganglion cell somata (Hidaka and Ishida, 1998; Kim and Rieke, 2003; Hayashida and Ishida, 2004). One would have expected the second spike to be smaller if the Na^+^ current that determined its rate of rise had not recovered from inactivation induced by the first spike. Therefore, the similarity of the spikes we examined suggests either that repetitive spiking does not cumulatively inactivate Na^+^ current in myelinated axons, or that effects of Na^+^ current inactivation might be seen at higher spike frequencies and/or during longer bursts of spikes. Recently, CaMKII has also been found to increase current through channels formed by Nav1.6 (Zybura et al., 2020). Because this is the Na^+^ channel isoform that populates optic nerve nodes of Ranvier (Boiko et al., 2001), the results reported and summarized above predict that activity-induced decreases in pT286 might decrease both Na^+^ and K^+^ conductances, and that both of these effects would tend to slow spike propagation.

During the past two dozen years, immunopositivities for, or pharmacological blockade profiles consistent with, five K^+^ channel subunits have been detected in adult rodent optic nerve (Baba et al., 1999; Rasband et al., 1999; Devaux et al., 2002, 2003, 2004; see also Klumpp et al., 1995). How these contribute to spike generation and propagation under various conditions and/or in different ganglion cell subtypes, remains to be further tested. The results reported here identify an additional K^+^ channel subunit in optic nerve and provide evidence that the ion channels it forms *in situ* are poised to be modulated by lighting conditions that alter CaMKII activation in retinal ganglion cell somata and axons (Ogata et al., 2012; Partida et al., 2018).

## Data availability statement

The original contributions presented in this study are included in the article/supplementary material, further inquiries can be directed to the corresponding author.

## Ethics statement

The animal study was reviewed and approved by the Animal Use and Care Administrative Advisory Committee of the University of California, Davis.

## Author contributions

AI: project conception and manuscript drafting (with details about methods from GO, GP, and AF). GO and GP: methodology. GO, GP, and AF: data collection. GO, GP, and AI: data analysis. GO: manuscript editing. All authors contributed to the article and approved the submitted version.

## Footnotes

1 http://imagej.net/Fiji, RRID:SCR_002285
